# Assessment of knowledge and attitude of healthcare professionals regarding the use of telemedicine: A cross-sectional study from rural areas of Sindh, Pakistan

**DOI:** 10.3389/fpubh.2022.967440

**Published:** 2022-10-26

**Authors:** Grouve Kumar, Harsha Komal Shardha, Waleed Tariq, Mansoor Ahmed Qazi, Kantesh Kumar, Chandni Maheshwari, Atif Hussain, Muhammad Junaid Tahir, Jharna Bai, Muhammad Sohaib Asghar

**Affiliations:** ^1^Gambat Medical College, Pir Abdul Qadir Shah Jeelani Institute of Medical Sciences (PAQSJIMS), Gambat, Pakistan; ^2^Peoples University of Medical and Health Sciences for Women, Nawabshah, Pakistan; ^3^Lahore General Hospital, Lahore, Pakistan; ^4^Department of Internal Medicine, Dow University of Health Sciences, Karachi, Pakistan; ^5^Shaikh Zayed Hospital, Lahore, Pakistan; ^6^Shaikh Khalifa Bin Zayed Al Nahyan Medical and Dental College, Lahore, Pakistan; ^7^Shaheed Mohtarma Benazir Bhutto Medical University, Larkana, Pakistan; ^8^Dow University of Health Sciences–Ojha Campus, Karachi, Pakistan

**Keywords:** remote consultation, telemonitoring, teleconsultation, COVID-19, rural

## Abstract

**Background:**

Telemedicine has proved its significance in the healthcare system, especially during the coronavirus disease 2019 (COVID-19) pandemic as it assists in the provision of early and effective healthcare to those in distant areas. This study aimed to assess the knowledge and attitude of healthcare professionals toward the use of telemedicine in the rural areas of Sindh, Pakistan.

**Materials and methods:**

The cross-sectional study was conducted with 212 healthcare professionals including physicians, consultants, surgeons, residents, dentists, and house officers working in public sector hospitals. The face-to-face method was used for data collection. Chi-square test and logistic regression analysis were applied to find the association between different variables and knowledge and practice of telemedicine using SPSS V 24.

**Results:**

The knowledge of participants regarding the usability of telemedicine and various tools used for the provision of medical services (video consultations, conferencing, use of applications, software, and online groups) was good. A total of 52.2% of participants showed agreement when they were questioned about being aware of online healthcare service provision. In this study, 52.8% of the participants practiced telemedicine by delivering healthcare services through online consultations. The majority of the participants were surgeons (25.9%), residents (23.1%), and physicians (18.9%). The internet (42.4%) and public media (25.9%) were the major sources of information for telemedicine. A total of 70.5% of participants suggested that strategies and efforts should be directed toward the enhanced implementation of telemedicine. Training in telemedicine as a source of information [OR = 13.02 (95% CI = 3.9–43.6), *p* < 0.001)] and awareness regarding the effect of telemedicine in healthcare quality [OR = 10.25 (95% CI = 2.9–35.4), *p* < 0.001)] remained the predicting factors for the practice of telemedicine among healthcare workers using multivariate regression analysis.

**Conclusion:**

Telemedicine has technologically revolutionized the medical sciences worldwide. The awareness level and usage of telemedicine were good among healthcare professionals in rural Sindh. Telemedicine should be utilized to provide quality healthcare in underprivileged areas by investing in infrastructure and education.

## Introduction

The use of telemedicine during the devastating period of the coronavirus disease 2019 (COVID-19) pandemic is remarkable (1). During the pandemic, people could not meet their regular follow-ups and faced difficulties in monitoring their chronic illnesses, i.e., diabetes mellitus, rheumatoid arthritis, hypertension, and oncology-related complications, as access to the outpatient department (OPD) was nearly impossible (2). Telemedicine has become apparent to be one of the swiftest and safest modes of providing health care assistance (3).

Telemedicine has unfolded its roots in almost every country of the world, from highly developed countries such as the USA and Europe to the sub-Saharan villages of Africa (4). Among the developed nations, many areas of health care are well-equipped with the use of telemedicine, while it has got inconsequential growth in many regions of lower-middle-income countries.

Telemedicine brings a blanket of hope to underprivileged areas. In general, these locations remain underserved as they lack the presence of working professionals (5). By serving economically backward areas and attempting to reverse the “brain drain” or flight of human capital, telemedicine not only augments the accessibility and quality of health but also aids healthcare workers to avail advantage of opportunities for professional growth (5).

While telemedicine is proliferating significantly and the developed nations report cherishing the exclusive benefits of this advancement, the question arises, "What is the standpoint of Pakistan regarding telemedicine?” World Health Organization (WHO) conducted a survey on telemedicine in 2016, which provides data on the unavailability of laws regarding telemedicine (6). Thousands of doctors are registered with the platforms, providing consultations to a great number of populations. Every minute, five patients search for expert medical consultation on online forums (7, 8). The developed countries are much better at providing telemedicine services and are thriving.

However, in Pakistan, people do not have any access to these advanced services provided through the telemedicine systems such as online healthcare providing forums. Though telemedicine is not a new entity, its progress in the interior of Sindh, Pakistan is not worth appreciating. Multiple factors such as lack of infrastructure, doctor and patient resistance to the latest advancements, inadequate financial resources, and poor training in information and communication technology hinder the growth of telemedicine system practices in Pakistan (9, 10). Therefore, our study aimed to assess the knowledge and attitude of healthcare professionals toward the use of telemedicine and the future opportunities in providing health services in the rural areas of Sindh, Pakistan.

## Materials and methods

A total of 212 healthcare professionals including physicians, consultants, surgeons, residents, dentists, and house officers participated in this study and filled out the questionnaire that was adapted from an Ethiopian research study (4). PUMHS data were collected from Peoples University of Medical & Health Sciences, Nawabshah, Sindh (PUMHS) and Pir Abdul Qadir Shah Jeelani Institute of Medical Sciences, Gambat, Sindh (PAQSJIMS). PUMHS is a public sector institute with facilities for both undergraduate and postgraduate medical education exclusively for females. It is an 875-bed hospital with multiple medical specialties. PAQSJIMS is the largest center of liver transplant in the country (300-bed) and also serves patients with other organ transplants and healthcare facilities.

The questionnaire comprised three main sections. The first section was comprised of sociodemographic data such as age, gender, hospital name, educational status, designation as a healthcare professional, and years of working experience. The next section of the questionnaire assessed the familiarity of the participants with telemedicine, its previous use, the source of information about telemedicine, awareness about its components, effect on healthcare quality, cost, and physician's time. The last section was modeled to know the opinions of the participants regarding the advantages, compatibility, and complexity of telemedicine as well as trial and observable abilities of the participants toward telemedicine.

Data were collected through an adapted questionnaire from an Ethiopian study (4). The face-to-face approach was used. The aim of the study was explained to the participant, and verbal instructions were provided. Informed consent was taken before initiating the survey. Anonymity was ensured with the responses of the participants at the time of consent. Ethical perspectives were followed as suggested by Helsinki Declaration, and the formal ethical approval for the study was taken from the PAQSJIMS, Gambat. (PAQSJIMS/GMC/1382).

The sample size was calculated *via* World Health Organization (WHO) sample size calculator. Response distribution was kept at 50% with a confidence interval of 95%, resulting in a sample size of 208. For data analysis, data were first entered in Microsoft Excel and subsequently imported into SPSS V.24. Descriptive statistics were applied for numerical and categorical variables. Chi-square test and logistic regression analysis were applied to find the association between different variables and knowledge and practice of telemedicine. The significance of the odds ratio (OR) from univariate analyses and adjusted OR (AOR) in multivariate analyses was assessed, accompanied by 95% confidence intervals (CI). A *p*-value of < 0.05 was considered statistically significant.

## Results

The study included 212 participants with a response rate of 92%. Of which, 152 (71.7%) were men and 60 (28.3 %) were women. A total of 89 (42%) participants belonged to the 23–32 years age group. The professional experience of 95 (44.8%) participants was <5 years, 51 (24.1%) was of 5–10 years, and 66 (31.1%) was more than 10 years. The majority of the participants were surgeons (55, 25.9%), residents (49, 23.1%), and physicians (40, 18.9%) (Table 1).

**Table 1:** Descriptive statistics for hospital name, gender, age distribution, and professional experience of the participants.

**Variables**	***N* (%)**
Total	212 (100%)
**Hospital name**
PUMHS	119 (56.1%)
PAQSJIMS	93 (43.9%)
**Gender distribution**
Males	152 (71.7%)
Females	60 (28.3%)
**Age distribution (years)**
23–32	89 (42%)
33–42	79 (37.3%)
43–52	30 (14.2%)
53 and above	14 (6.6%)
**Professional experience (years)**
<5	95 (44.8%)
5–10	51 (a24.1%)
>10	66 (31.1%)
**Designations of healthcare professionals**
Anesthesiologist	5 (2.4%)
Dentist	4 (1.9%)
Family counselor	1 (0.5%)
House officer	11 (5.2%)
Medical officer	31 (14.6%)
Neurologist	1 (0.5%)
Oncologist	2 (0.9%)
Psychiatrist	1 (0.5%)
Physician	40 (18.9%)
Pulmonologist	2 (0.9%)
Radiologist	4 (1.9%)
Resident	49 (23.1%)
Senior medical officer	6 (2.8%)
Surgeon	55 (25.9%)

A total of 112 (52.8%) participants had practiced telemedicine. The internet (90, 42.4%) and public media (55, 25.9%) were the major sources of information for telemedicine. As for the tools of the era of telemedicine, teleconsultation (120, 56.6%) was the most known and had the least knowledge about teleporting (1, 0.47%) (Table 2).

**Table 2:** Knowledge about telemedicine.

**Variables**		***N* (%)**
Telemedicine ever	Yes	112 (52.8%)
practiced	No	100 (47.1%)
Source of information	Training	36 (16.9%)
regarding telemedicine	Public media	55 (25.9%)
	Internet	90 (42.4%)
	Colleague	29 (13.6%)
	Other	2 (0.94%)
Knowledge about the	Teleconferencing	8 (3.77%)
tools of telemedicine	Teleconsultation	120 (56.6%)
	Teleconsultation and teleconferencing	25 (11.7%)
	Teleconsultation and telesurgery	15 (7.07%)
	Tele reporting	1 (0.47%)
	Telesurgery	7 (3.30%)
	Telesurgery, teleconsultation, and teleconferencing	18 (8.49%)
	Don't know	18 (8.49%)

A total of 187 (88.2%) participants were aware of the effect of telemedicine on healthcare. A majority of participants believed that telemedicine can reduce the need for medical staff (150, 70.7%) and unnecessary transportation costs (189, 89.1%). According to 183 (86.3%) participants, telemedicine is time-saving for clinicians or healthcare professionals (Table 3).

**Table 3:** Awareness about the effect of telemedicine.

**Variables**		***N* (%)**	***p*-value**
Are you aware of the effect of	Yes	187 (88.2%)	0.65
telemedicine on healthcare quality?	No	15 (7.07%)	
Do you feel telemedicine can reduce	Yes	150 (70.7%)	0.58
the need for medical staff?	No	62 (29.2%)	
Do you believe telemedicine can reduce	Yes	189 (89.1%)	0.35
unnecessary transportation costs?	No	23 (10.8%)	
Do you think that telemedicine is	Yes	183 (86.3%)	0.48
time-saving for clinicians or healthcare professionals?	No	29 (13.6%)	

The respondents agreed that telemedicine can increase communication among healthcare providers (141, 66.5%), it can reduce the number of visits to healthcare centers (151, 71.2%), enable the accomplishment of tasks more quickly (118, 55.6%), improve clinical decisions (67, 31.6%), and provide more comprehensive healthcare services (83, 39.15%) (Table 4).

**Table 4:** Attitudes of healthcare professionals regarding the advantages of telemedicine.

**Variables**	**Strongly disagree**	**Disagree**	**Neutral**	**Agree**	**Strongly agree**
	***N* (%)**	***N* (%)**	***N* (%)**	***N* (%)**	***N* (%)**
Reduce medical errors	20 (9.4%)	84 (39.6%)	55 (25.9%)	46 (21.6%)	7 (3.30%)
Facilitate diagnosis and treatment	4 (1.88%)	35 (16.5%)	29 (13.6%)	132 (62.2%)	12 (5.66%)
Increase communication among health care providers	1 (0.47%)	16 (7.54%)	25 (11.7%)	141 (66.5%)	29 (13.6%)
Telemedicine can reduce the number of visits to healthcare centers	4 (1.88%)	8 (3.77%)	15 (7.07%)	151 (71.2%)	34 (16.0%)
Enables me accomplish my task more quickly	4 (1.88%)	23 (10.8%)	54 (25.4%)	118 (55.6%)	13 (6.13%)
Improve clinical decisions	10 (4.71%)	59 (27.8%)	59 (27.8%)	67 (31.6%)	17 (8.01%)
Provide more comprehensive healthcare services	5 (2.35%)	47 (22.16%)	61 (28.77%)	83 (39.15%)	15 (7.07%)

Only 65 (30.6%) of participants agreed that telemedicine is compatible with all aspects of their work and with the current situation of 98 (46.2%) of the participants. Similarly, they agreed that it fits with the way they like to work and their working style of 74 (34.9%) and 69 (32.5%) participants, respectively. For the complexity of telemedicine, many disagreed that learning to operate it is hard (91, 42.9%), increases staff workload (128, 60.3%), and threatens information confidentiality and patient privacy (85, 40.0%) (Table 5).

**Table 5:** Attitudes of healthcare professionals regarding the compatibility and complexes of telemedicine.

**Variables**	**Strongly disagree**	**Disagree**	**Neutral**	**Agree**	**Strongly agree**	**Total mean**
	***N* (%)**	***N* (%)**	***N* (%)**	***N* (%)**	***N* (%)**	**score**
**Compatibility**						
Telemedicine is compatible with all aspects of my work.	8 (3.77%)	64 (30.1%)	66 (31.1%)	65 (30.6%)	9 (4.24%)	3.0
Telemedicine is completely compatible with my current situation	12 (5.66%)	46 (21.6%)	44 (20.7%)	98 (46.2%)	12 (5.66%)	
Telemedicine fits well with the way I like to Work	12 (5.66%)	61 (28.7%)	53 (25.0%)	74 (34.9%)	12 (5.66%)	
Using telemedicine fits well to my working style	13 (6.13%)	62 (29.2%)	60 (28.3%)	69 (32.5%0	8 (3.77%)	
**Complexity**						
Telemedicine requires a lot of mental effort.	5 (2.35%)	55 (25.9%)	56 (26.4%)	80 (37.7%)	16 (7.54%)	2.8
Learning to operate telemedicine is hard.	22 (10.3%)	91 (42.9%)	57 (26.8%)	38 (17.9%)	4 (1.88%)	
Telemedicine increases staff work load.	25 (11.7%)	128 (60.3%)	36 (16.9%)	18 (8.49%)	5 (2.35%)	
Telemedicine creates new responsibilities for healthcare professionals.	9 (4.24%)	34 (16.0)	34 (16.0)	122 (57.5%)	13 (6.13%0	
Telemedicine threatens information confidentiality and patient privacy.	20 (9.43%)	85 (40.0%)	42 (19.8%)	54 (25.4%)	11 (5.18%)	

Table 6 shows that regarding trialability, participants agreed that it is a great opportunity for healthcare professionals (127, 59.9%), takes very much effort to try out (99, 46.6%), and application on a trial basis is enough to see what it could do (125, 58.9%), and they would like to try out telemedicine applications before using it (135, 63.6%). A total of 81 (38.2%) participants agreed that they observed what other hospital staff do with telemedicine, and 72 (33.9%) participants had observed telemedicine being used for many tasks in the hospital. In total, 79 (37.2%) participants disagreed about the visibility of telemedicine in hospitals where they work. Training in telemedicine as a source of information [OR = 13.02 (95% CI = 3.9–43.6), *p* < 0.001)] and awareness regarding the effect of telemedicine in healthcare quality [OR = 10.25 (95% CI = 2.9–35.4), *p* < 0.001)] remained the predicting factors for the practice of telemedicine among healthcare workers regardless of their gender, age, designation, education level, institution, and years of experience as shown in Table 7.

**Table 6:** Attitudes of healthcare professionals regarding trialability and observability for telemedicine.

**Variables**	**Strongly disagree**	**Disagree**	**Neutral**	**Agree**	**Strongly agree**	**Total mean**
	***N* (%)**	***N* (%)**	***N* (%)**	***N* (%)**	***N* (%)**	**score**
**Trial ability**						
Telemedicine application is a great opportunity for healthcare professionals.	7 (3.30%)	14 (6.60%)	39 (18.3%)	127 (59.9%)	25 (11.7%)	3.4
Take very much effort to try out Telemedicine.	6 (2.83%)	47 (22.1%)	52 (24.5%)	99 (46.6%)	8 (3.77%)	
Using telemedicine on a trial basis is enough to see what it could do.	8 (3.77%)	30 (14.1%)	39 (18.3%)	125 (58.9%)	10 (4.71%)	
Would like to try out telemedicine applications before using it.	9 (4.24%)	20 (9.43%)	37 (17.4%)	135 (63.6%)	11 (5.18%)	
**Observability**						
What other hospital staffs do with Telemedicine?	12 (5.66%)	50 (23.5%)	56 (26.4%)	81 (38.2%)	13 (6.13%)	2.9
Telemedicine quite visible in the Hospital where I work.	21 (9.90%)	79 (37.2%)	44 (20.7%)	64 (30.1%)	6 (2.83%)	
In the Hospital, I see telemedicine being used for many tasks.	26 (12.2%)	57 (26.8%)	46 (21.6%)	72 (33.9%)	11 (5.18%)	

**Table 7:** Association of general characteristics of the study population with the practice of telemedicine.

**Variables**		**OR (95% CI)**	***P*-value**	**aOR (95% CI)**	***P*-value**
Gender	Male	1.29 (0.71–2.34)	0.410	1.13 (0.53–2.42)	0.756
	Female	1.00	–	1.00	–
Age distribution (years)	23–32	1.02 (0.33–3.16)	0.969	1.40 (0.26–7.58)	0.694
	33–42	1.03 (0.33–3.20)	0.965	1.09 (0.30–4.03)	0.892
	43–52	2.00 (0.55–7.29)	0.294	1.48 (0.35–6.27)	0.596
	53 and above	1.00	–	1.00	–
Year of experience	<5 years	1.00	–	1.00	–
	5–10 years	0.98 (0.50–1.94)	0.958	0.98 (0.36–2.71)	0.886
	>10 years	1.57 (0.83–2.97)	0.164	1.04 (0.31–3.21)	0.995
Hospital name	PAQSJIMS (GIMS Hospital)	0.67 (0.39–1.16)	0.155	0.55 (0.27–1.14)	0.107
	PUMHS (PMC Hospital)	1.00	–	1.00	–
Education level	Graduate level (MBBS, BDS)	1.00	–	1.00	–
	Postgraduate	1.42 (0.82–2.46)	0.206	1.72 (0.54–5.44)	0.357
Designation	Trainee	1.00	–	1.00	–
	Physician	1.70 (0.80–3.61)	0.167	1.35 (0.38–4.81)	0.640
	Surgeon	1.14 (0.59–2.21)	0.701	1.10 (0.32–3.86)	0.878
	Allied Sciences	1.02 (0.39–2.67)	0.967	0.94 (0.22–3.93)	0.932
Source of information regarding Telemedicine	Training	13.02 (3.9–43.6)	**<0.001**	10.05 (2.9–35.2)	**<0.001**
	Public Media	1.63 (0.65–4.09)	0.302	1.48 (0.54–4.10)	0.447
	Internet	2.29 (0.97–5.42)	0.058	2.16 (0.86–5.45)	0.103
	Colleagues	1.00	–	1.00	–
Are you aware of the effect of Telemedicine on	Yes	10.25 (2.9–35.4)	**<0.001**	7.63 (2.13–27.3)	**0.002**
healthcare quality?	**No**	1.00			

OR, odds ratio; aOR, adjusted odds ratio; CI, confidence interval.

## Discussion

Telemedicine is revolutionizing healthcare worldwide by combining traditional medicine with advanced telecommunication. It has improved access to standard and specialized medical management in underdeveloped areas. This study assessed the attitude and perceptions of healthcare professionals toward telemedicine in the rural areas of Sindh, Pakistan.

The awareness level about the use of telemedicine services provided by various tools, such as video conferencing and video consultations, is 52.2%. Zayapragassarazan et. al reported that 41% of respondents have good knowledge of telemedicine in India (11). Elhadi et. al found that 56% of healthcare workers have a high awareness level of telemedicine that is comparable to the current study (12). The contrast and comparison between Zayapragassarazan et. al and Elhadi et. al, respectively, can be attributed to exposure to COVID-19. The COVID-19 pandemic has raised the awareness and application of telemedicine as the lockdown conditions had restricted movements. Telemedicine facilitates access to medical advice and approach for patients despite limited mobility (13, 14).

The Internet is the major source of information about telemedicine (42.4%). The internet has become a major source of information in the modern era as humans rely increasingly on the internet and social media for routine social, financial, and healthcare activities (15).

The attitude toward telemedicine is reported positive as it facilitates diagnosis and treatment (67.8%), increases communication among healthcare providers (70.1%), and reduces the number of visits to healthcare centers (87.2%). Telemedicine is believed to be effective in faster medical care, better care in remote areas, and reduces budgetary needs (16, 17). Telemedicine, being an advanced innovation, has proven its significance since its introduction in healthcare, resulting in the positive attitude of both healthcare providers and patients. The majority of the respondents believed that telemedicine does not threaten the confidentiality and privacy of the patient. Previously, varied responses were reported that telemedicine services threaten the privacy of patients, as unauthorized persons may access the data of the patients by using the smart modes of technology and may jeopardize the confidentiality of the patient (18). Furthermore, telemedicine reduces the workload on staff (72%) as it prevents unnecessary hospital visits, hospital admissions, and emergency overuse ultimately lowering the burden on medical staff (19).

The participants had a negative attitude that telemedicine requires a lot of mental effort (45.2%) and creates new responsibilities for healthcare professionals (63.6%). Despite the presence of telemedicine in healthcare for over decades, it has not been used to its full potential to serve mankind. Multiple barriers, such as lack of updated technology, formal organizational structure, appropriate training, rules and regulations, and poor financial resources, hinder the development of telemedicine health delivery system in underdeveloped countries (20, 21).

## Limitations

There are certain limitations to this study. First, it was conducted in two tertiary care, public sector hospitals in the interior of Sindh, thus a considerable proportion of the healthcare professionals working in the other rural areas of Sindh and other provinces were not involved in this study. Therefore, the results could not be generalized to the larger population of rural Sindh or the whole Pakistani population. Second, because this is a cross-sectional study, we could not establish causal inferences. The third limitation is that recall bias could not be ignored.

## Conclusion

Telemedicine is influencing healthcare worldwide and is technologically revolutionizing the medical sciences. Most of the participants had a high awareness level of telemedicine, and a positive attitude was reported about the multiple aspects and benefits of telemedicine in the rural areas of Sindh. Telemedicine has a massive influence on healthcare as it has improved access to quality medical care, reduced the cost of medical treatment, and overcome geographical and time barriers. Telemedicine can fill the shortage of resources and poor doctor-to-patient ratio. International and national healthcare authorities should collaborate to develop the infrastructure at national, provincial, and local levels and should provide standard health services to humanity.

## Data availability statement

The original contributions presented in the study are included in the article/supplementary files, further inquiries can be directed to the corresponding author/s.

## Ethics statement

The studies involving human participants were reviewed and approved by Formal ethical approval for the study was taken from the PAQSJIMS, Gambat (PAQSJIMS/GMC/1382). The patients/participants provided their written informed consent to participate in this study.

## Author contributions

MQ conceived the idea. GK and HS collected the data. MQ, MA, GK, and HS analyzed and interpreted the data. HS, KK, CM, MA, GK, WT, AH, and MT did write up of the manuscript. MA, JB, MT, and WT reviewed the manuscript for intellectual content critically. All authors approved the final version of the manuscript.

## Conflict of interest

The authors declare that the research was conducted in the absence of any commercial or financial relationships that could be construed as a potential conflict of interest.

## Publisher's note

All claims expressed in this article are solely those of the authors and do not necessarily represent those of their affiliated organizations, or those of the publisher, the editors and the reviewers. Any product that may be evaluated in this article, or claim that may be made by its manufacturer, is not guaranteed or endorsed by the publisher.
